# Toll-like receptor expression in human non-small cell lung carcinoma: potential prognostic indicators of disease

**DOI:** 10.18632/oncotarget.19463

**Published:** 2017-07-22

**Authors:** Alison K. Bauer, Brad L. Upham, Elizabeth A. Rondini, Meredith A. Tennis, Kalpana Velmuragan, David Wiese

**Affiliations:** ^1^ Department of Environmental and Occupational Health, University of Colorado Anschutz Medical Campus, Aurora, CO 80045, USA; ^2^ Department of Pediatrics and Human Development, Michigan State University, East Lansing, MI 48824, USA; ^3^ Department of Pathobiology and Diagnostic Investigation, Michigan State University, East Lansing, MI 48824, USA; ^4^ Department of Pulmonary Sciences and Critical Care Medicine, University of Colorado Anschutz Medical Campus, Aurora, CO 80045, USA; ^5^ McLaren Regional Medical Center, Flint, MI, 48532, USA

**Keywords:** adenocarcinoma, innate immunity, non-small cell lung carcinoma, prognostic, toll-like receptor

## Abstract

**Introduction:**

Lung cancer remains the highest cause of cancer mortality worldwide. Toll-like receptors (TLR) are innate immune receptors that have both pro- and anti-tumorigenic properties. Based on findings from epidemiological studies and in rodents, we hypothesized that elevated TLR expression would be a positive prognostic indicator of disease in non-small cell lung carcinoma patients.

**Results:**

Higher mRNA expression of TLR1-3 and 5-8 were significantly associated with increased overall survival (OS) when analyzed individually or as a group in both non-small cell lung carcinoma (NSCLC) patients and in the adenocarcinoma (ADC) subtype. Significant co-expression of many TLR combinations in ADC patients were also observed via RNA sequencing. Immunostaining demonstrated TLR4 and 8 significantly correlated in tumor tissue, similar to RNA.

**Methods:**

We used kmplot.com to perform a meta-analysis on mRNA expression of TLR1-10 to determine any significant associations with OS in NSCLC and the ADC subtype. cBioportal was also used simultaneously to assess co-expression in TLR1-10 in ADC patients via RNA sequencing and to identify any molecular alterations. Lastly, immunostaining for a subset of TLRs was conducted on ADC patients.

**Conclusions:**

Expression of innate immune receptors TLR1-10 is associated with improved survival outcomes in NSCLC. Thus, further evaluation of their predictive capacity and therapeutic utility is warranted.

## INTRODUCTION

One out of every four cancers is lung cancer; thus, lung cancer is among those cancers where early detection and prevention is critical [1]. Human lung cancer includes small cell lung carcinoma (SCLC) and non-small cell lung carcinoma (NSCLC) types, with NSCLC accounting for 85% of disease. NSCLC is divided into several subtypes, including adenocarcinoma (ADC), squamous, and large cell carcinoma, of which ADC has the highest incidence among smokers and is the typical subtype found in non-smokers [2]. Several recent advances in lung cancer detection have been made, such as the use of CT scans in high risk individuals [3] and genetic analyses [4], although genetic analyses are still largely investigational. Determining novel biomarkers found at the initial stages of lung cancer can contribute to early detection, which is a focus of this translational study.

Inflammatory mediators produced in situ by activated innate cells are indispensable to immunity during injury or infection, but are also associated with increased risks of cancer and tumorigenesis [5]. Thus, understanding the precise roles of inflammatory mediators in cancer could serve as potential biomarkers for early detection of cancers. In NSCLC, the role for the innate immune system is controversial due to evidence for both pro- and anti-inflammatory effects, thus, the pathways mediating these mechanisms need further elucidation [6–10]. The most commonly studied receptors in innate immunity are the toll-like receptors (TLRs). Eleven human TLRs exist, although only TLR1-9 are conserved among humans and rodents [11]. TLR4 was the first human homolog to TOLL receptor in Drosophila melanogaster identified and the most commonly studied TLR based on its ligand lipopolysaccharide (LPS, a component of endotoxin) [12, 13]. TLR4 is triggered by the binding of activated host ligand(s) to form homodimers at the cell surface [14], which leads to the recruitment of adaptor molecules to the cytoplasmic domain, such as MYD88 and TRIF [15]. Downstream effectors from the TLR4/MYD88 signaling cascade include activation of NFκB leading to cytokine production and other functions as well as mitogen activated protein kinases (MAPK) such as ERK1/2 and p38 leading to additional transcription factor activation, such as CREB [16, 17]. All 11 human TLRs have known exogenous ligands called pathogen-associated molecular patterns (PAMPs), except TLR10, which are shared by many pathogens but not expressed by the hosts [18]. Danger -associated molecular patterns (DAMPs) are endogenous ligands of TLRs, which are released from stressed cells undergoing necrosis, that promote inflammatory responses [18]. TLRs are expressed on many cell types, including macrophages, dendritic cells (DC)s, lymphocytes, and respiratory epithelial cells [19, 20].

Activation of innate immunity through TLR4 can both exacerbate [13, 21, 22] and inhibit [23–25] lung inflammation and injury. Interestingly, several epidemiological studies observed significant decreases in lung cancer risk in individuals exposed to endotoxin (LPS), such as farm and some textile workers [26, 27]. TLR4 is the primary receptor that binds endotoxin [13] and, thus, is likely involved in the protective effect observed with endotoxin exposure. Consistent with this, Tlr4-deficient mice are also significantly more sensitive to lung tumor promotion, pulmonary inflammation, and have demonstrated differences in immune cell profiles compared to wild-type mice in a primary ADC model [28–30]. Protective effects of other TLRs on lung, prostate, gastric, and breast cancers have also been reported in humans and in mouse models [27–45]. Therefore, we hypothesized that the mRNA expression of TLRs 1-10 would be a positive prognostic indicator for lung cancer risk. We first examined TLR1-10 mRNA expression profiles using a meta-analysis tool (kmplot.com) and compared expression levels to overall survival (OS), first progression (FP), and post-progression survival (PPS). Co-expression patterns for TLR1-10 mRNA expression were simultaneously identified using the cBioportal database (http://cBioportal.org) and by immunohistochemical (IHC) staining for several of these TLRs. This is the first combined analysis of human TLR's 1-10 in NSCLC, with a focus on ADC.

## RESULTS

This study presents results first indicating which TLRs are associated with improved outcomes for NSCLC and ADC patients, followed by more specific data on the co-expression of TLRs and their molecular alterations in ADC patients. Further analyses determined the types of TLR positive cell types observed in the tumor tissue as well as the correlative relationships between the various TLRs in the same patients.

### Several TLRs are associated with improved outcomes in NSCLC and ADC patients

**Figure 1:**
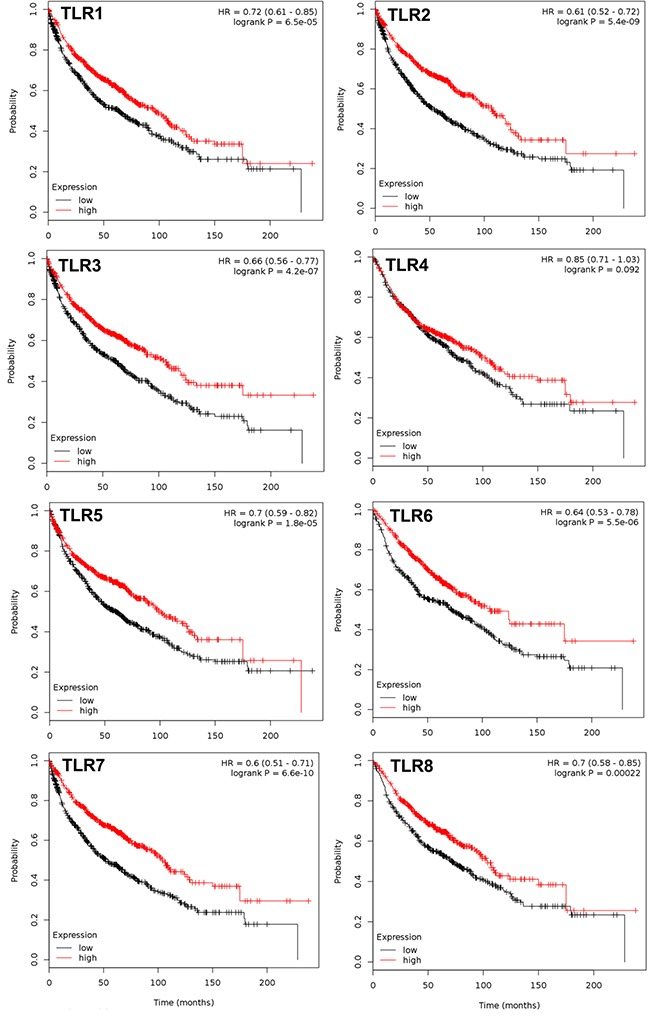
TLRs that significantly associate with overall survival in NSCLC patients Multivariate analysis was performed (co-variates: gender and histology) and TLRs that significantly associate with overall survival (OS) are presented with the exception of TLR4. N= 1926 for every TLR except TLR4 (n=1587); analyzed via kmplot.com. Hazard ratio (HR), 95% confidence intervals, and logrank P presented per TLR.

**Figure 2:**
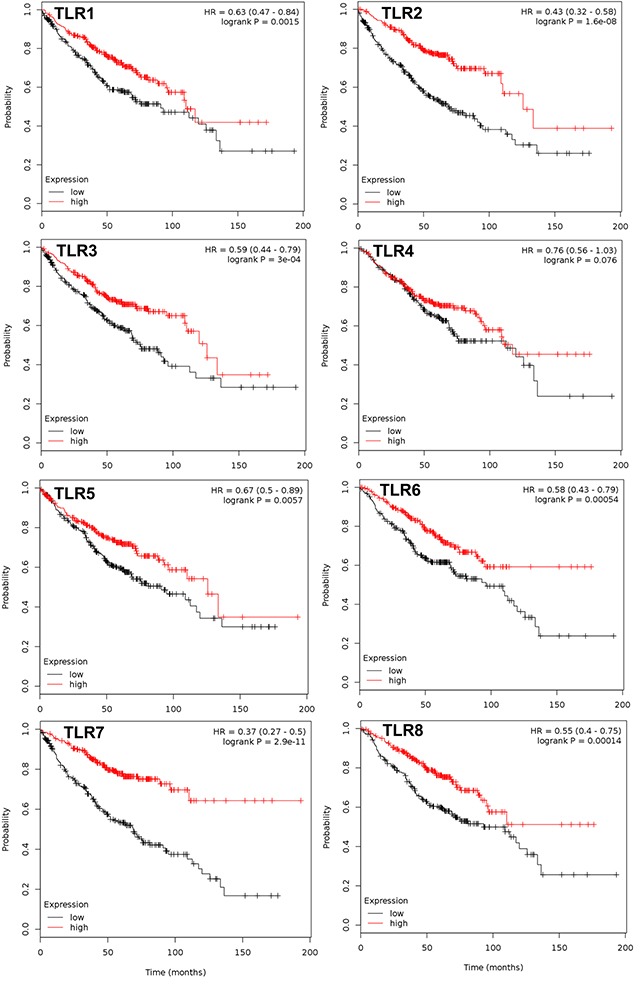
TLRs that significantly associate with overall survival in the ADC subtype Multivariate analysis was performed (co-variates: gender and stage) and TLRs that significantly associate with overall survival (OS) are presented with the exception of TLR4. N= 720 for every TLR except TLR4 (n=673); analyzed via kmplot.com. Hazard ratio (HR), 95% confidence intervals, and logrank P presented per TLR.

**Figure 3:**
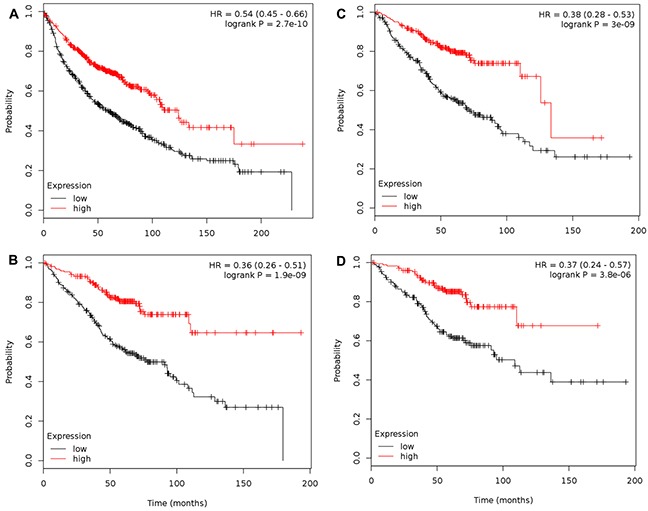
Combination of TLR1-10 significantly associates with overall survival for both NSCLC and ADC, specifically in stage 1 patients **(A)** Association between NSCLC patients and TLR1-10 mRNA expression (n= 1145) via multivariate analysis (co-variates: gender and histology); **(B)** Association between NSCLC patients that are only stage 1 and TLR1-10 mRNA expression (n=449) via multivariate analysis (co-variates: gender and histology); **(C)** Association between ADC patients and TLR1-10 mRNA expression (n=673) via multivariate analysis (co-variates: gender and stage); **(D)** Association between ADC patients that are only stage 1 and TLR1-10 mRNA expression (n=346) via univariate analysis. Analysis was performed using kmplot.com and Supplementary Table 1 has details for each of these analyses as well as stage 2 analyses. Hazard ratio (HR), 95% confidence intervals, and logrank P presented.

All 10 TLRs with available probes were compared individually and then combined for any significant associations with OS, PPS, and FP using kmplot.com. Table 1A displays all the individual TLRs, the univariate p values, multivariate p values for gene, histology, and gender, and hazard ratio's (HR) in all NSCLC patients for OS. Figure 1 depicts the genes with significantly improved OS (TLR1,2, 3, 5, 6, 7, and 8), or in other words high TLR expression associated with increased survival with HR all below 1. For each one, both histology and gender are also significant when considered as co-variates in the multivariate analysis. Similar trends were observed when we considered the most prevalent subtype of NSCLC, ADC (Figure 2). TLR1, 2, 3, 5, 6, 7 and 8 were individually significantly associated with OS in ADC patients. When these same TLRs were stratified by stage, but not gender, they also associated as a co-variate in the multivariate analyses (see Table 1B). We also included TLR4 on these graphs due to previous studies in our laboratory as well as additional findings from others [28, 30, 36, 43]. We then compared all TLRs (1-10) combined (multi-gene) and the TLRs that were significantly associated to OS individually (TLR1,2,3,5,6,7,8) combined to determine if the combination of TLRs improved the OS. Figure 3 and Supplementary Table 1 demonstrate that TLR1-10 combined in the analysis are highly significantly associated with improved survival in both NSCLC (HR = 0.54, *P* = 2.7 × 10^−10^) and ADC patients (HR = 0.38, *P* = 3.0x 10^−9^). The analysis was similar if we only considered those TLRs that were significant individually (TLR1,2,3,5,6,7,8: NSCLC, HR=0.52, *P* = 2 × 10^−11^; ADC, HR=0.38, *P* = 2 × 10^−9^; see Supplementary Table 1). Since the analysis was weighted based on 1/HR per gene, these results suggest that the combined analysis is driven largely by those TLRs with the lowest HR.

**Table 1A:** Meta-analysis results for associations between the TLRs and overall survival for NSCLC patients.^*^

			Univariate		Multivariate			
Gene	Affy Probe ID	n	*P* value	HR^	*P* value (gene)^**^	HR (gene)	*P* value (histology)	*P* value (gender)
TLR1	210176_at	1926	6.50E-05	0.72 (0.61-0.85)	0.003	0.74 (0.63 0.87)	1.00E-15	0.0004
TLR2	204924_at	1926	5.40E-09	0.61 (0.52-0.72)	6.00E-04	0.74 (0.62-0.88)	1.00E-15	0.0006
TLR3	206271_at	1926	4.20E-07	0.66 (0.56-0.77)	0.0052	0.78(0.66-0.93)	1.00E-15	0.0003
TLR4	232068_s_at	1587	ns	0.85 (0.71-1.03)	ns	-	-	-
TLR5	210166_at	1926	1.80E-05	0.7 (0.59-0.82)	0.0173	0.81 (0.68 0.96)	1.00E-15	0.0005
TLR6	239021_at	1926	5.50E-06	0.64 (0.53-0.78)	0.001	0.72 (0.59 0.87)	1.00E-15	0.0017
TLR7	220146_at	1926	6.60E-10	0.6 (0.51-0.71)	1.00E-15	0.6 (0.51-0.71)	1.00E-15	0.0006
TLR8	229560_at	1587	0.00022	0.7 (0.58-0.85)	0.0095	0.78 (0.64-0.94)	1.00E-15	0.0024
TLR9	223903_at	1587	ns	0.94 (0.78-1.13)	ns	-	-	-
TLR10	223751_x_at	1926	ns	0.85 (0.71-1.03)	ns	-	-	-

^*^ For all significant TLRs, FDR 5% was tested, NSCLC combined samples *P* <0.0173; ADC samples *P* <0.0066; ^**^ Multivariate for all NSCLC for gender and histology; ^ Hazard ratio. Ns, not significant. Dash indicates test was not performed due to non-significance in univariate analysis.

**Table 1B:** Meta-analysis results for associations between the TLRs and overall survival for ADC patients.^*^

			Univariate		Multivariate			
Gene	Affy Probe ID	n	*P* value	HR^	*P* value (gene)^**^	HR (gene)	*P* value (stage)	*P* value (gender)
TLR1	210176_at	720	1.50E-03	0.63 (0.47-0.84)	0.0029	0.65 (0.48 - 0.86)	1.00E-15	ns
TLR2	204924_at	720	1.60E-08	0.43 (0.32-0.58)	1.00E-15	0.45 (0.33-0.6)	1.00E-15	ns
TLR3	206271_at	720	3.00E-04	0.59 (0.44-0.79)	0.0028	0.64 (0.48-0.86)	1.00E-15	ns
TLR4	232068_s_at	673	ns	0.76 (0.56-1.03)	-	-	-	-
TLR5	210166_at	720	0.0057	0.67 (0.5-0.89)	0.0066	0.67 (0.5 - 0.89)	1.00E-15	ns
TLR6	239021_at	720	0.00054	0.58 (0.43-0.79)	0.0049	0.64 (0.46 - 0.87)	1.00E-15	ns
TLR7	220146_at	720	2.90E-11	0.37 (0.27-0.5)	1.00E-15	0.41 (0.3 - 0.57)	1.00E-15	ns
TLR8	229560_at	720	0.0027	0.69 (0.54-0.88)	0.0014	0.6 (0.43-0.82)	1.00E-15	ns
TLR9	223903_at	673	ns	1.17 (0.87-1.59)	ns	-	-	-
TLR10	223751_x_at	720	ns	0.97 (0.78-1.23)	-	-	-	-

^*^ For all significant TLRs, FDR 5% was tested, ADC combined samples *P* <0.0066; ^**^ Multivariate for ADC for gender and stage; ^ Hazard ratio. Ns, not significant. Dash indicates test was not performed due to non-significance in univariate analysis.

Interestingly, when only stage 1 patients were considered for combined TLR1-10 analysis (Figure 3B, n=449 for NSCLC; Figure 3D, n=346 for ADC), the HR were further decreased (NSCLC, HR = 0.36, *P* =1.9 × 10^−9^; ADC, HR =0.37, *P* = 3.8 × 10^−6^). The decrease in p value for stage 1 was largely due to decreased n, and thus power. The individual TLRs significant for stage 1 NSCLC were TLR2, 3, 5, 6, 7, 8 and for stage 1 ADC patients all TLR except TLR9 (see Supplementary Table 1C, 1D). Stage 2 patients did not associate in the NSCLC patients, but did when only ADC was considered, however there were few of these patients (NSCLC, n=161; ADC, n=168; see Supplementary Table 1A, 1B), thus these analyses are not as reliable. A lack of information existed for stage ≥ 3 to perform analyses.

TLR5, 6, and 7 also associated with improved outcomes using PPS with univariate analysis (TLR5, HR= 0.69 (0.54-0.89), *P* =0.0045; TLR6, 0.63 (0.41-0.97), *P* =0.034; TLR7, HR = 0.64 (0.5-0.83), *P* =5.3×10^−4^) however, following multivariate analysis, none remained significant due in part to a lack of power. However, combining all TLRs for the univariate analysis to determine associations with PPS was significant (HR= 0.58 (0.38-0.9), *P* =0.013) for NSCLC. ADC did not have enough samples to perform the analysis. Similarly, the FP analysis associated only with TLR2 for univariate analysis and following multivariate analysis (NSCLC, HR =0.63 (0.47-0.85), *P* =2.5×10^−3^; ADC, HR=0.55 (.38-.78), *P* =0.00085). When all TLRs were combined in a multivariate analysis, TLRs significantly associated with FP (HR= 0.72 (0.53-0.98), *P* =0.038) for NSCLC, but not ADC.

Lastly, using the kmplot.com website, we were also able to determine any significant differences between control, non-tumor bearing patients (n=86) and lung NSCLC patients (n=1926). Several TLRs had significantly lower mRNA expression in the control versus NSCLC tumor tissue, namely TLR1,2,3,7,9, and 10 while TLR4,5,6 and 8 did not (see Supplementary Table 2). No comparisons were available for ADC only.

### Co-expression of TLRs in ADC patients

**Figure 4:**
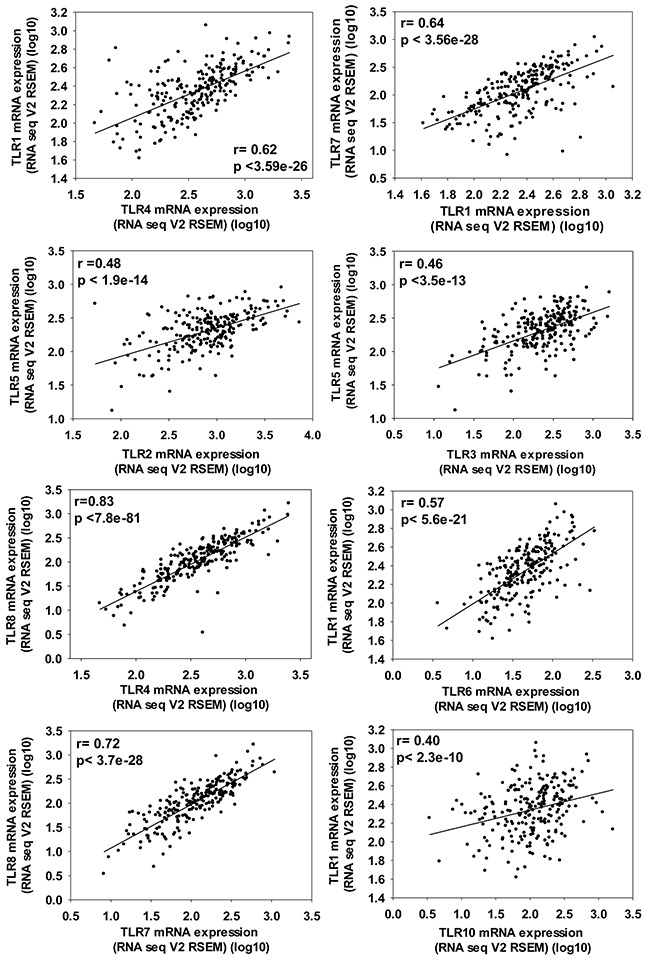
Significant co-expression between TLRs in ADC patients demonstrating the importance of evaluating multiple TLRs at once in a cancer population ADC patient data from TCGA provisional set (cBioportoal.org) that included n=230 samples analyzed by RNA sequencing and presented here as RNAseq V2 RSEM (RNA-Seq by Expectation Maximization) with the Pearson correlation as well as p value noted on each graph.

Analysis of co-expression of the different TLRs in lung ADC revealed that several of the TLRs were expressed similarly in ADC (see Supplementary Table 3). Figure 4 demonstrates the TLRs with the largest significant correlations, however, some TLRs co-express with multiple other TLRs (eg.TLR1 co-expresses with TLR4, 6, 7, and 10, see Figure 4). In an ADC cell line (NCI-H1650), the majority of the TLRs were expressed similarly (see Supplementary Figure 1). Additional genes that are also significantly co-expressed with each TLR are listed separately in Supplementary Table 3.

### Molecular alterations in TLRs in ADC patients

The cBioportal.org website was also used to evaluate molecular alterations for each TLR. The Pan-Lung Cancer panel (n=1089 patients both ADC and squamous cell carcinoma) demonstrated low somatic mutation, deletion and amplification rates for TLR1, 2, 6, 9, and 10, however for each of the other TLRs, one of these molecular alterations was > 2% ≤ 10%, see Supplementary Table 4A. When we compare these results to the same ADC panel used for the co-expression analysis, the numbers change slightly, but the trend remains similar. We also included mRNA upregulation in the ADC molecular alteration table (Supplementary Table 4C) that demonstrates that four TLRs (TLR4, 5, 6 and 9) were upregulated between 5-10%, but all were ≥ 3%.

### Clinical characteristics for the human tumors stained for TLRs

Table 2 displays the subtypes of ADC that were assessed in this pilot study (acinar, acinar mucinous, and acinar solid) with stage and principle inflammatory cell type noted. The types of inflammatory cells observed in the tumor tissue were primarily lymphocytes. Because the sample number is low for the different morphologies at each stage, true inflammation differences cannot be adequately determined.

**Table 2:** Clinical characteristics of human lung adenocarcinomas

ID	Stage^b^	Primary Growth Pattern	Principle Inflammatory Cell Type^a^
1	I	Acinar	L
2	I	Acinar	L
3	I	Acinar, mucinous	L,AM
4	II	Acinar	L
5	II	Acinar	L
6	II	Mucinous, solid	L
7	II	Acinar	AM
8	II	Acinar	L
9	II	Acinar, mucinous	L,AM
10	II	Acinar, solid	L
11	II	Acinar	L,AM
12	II	Acinar, mucinous	L
13	II	Acinar	L
14	II	Acinar	L
15	III	Solid	L
16	III	Acinar, solid	AM,L
17	III	Solid	L
18	III	Acinar, solid	L,AM

^a^. Principle inflammatory cell type present in associated tissue: L=lymphocytes; AM=alveolar macrophages; N= none observed.

^b^. Tumor Staging based on the Pathologic TNM classification.

### Expression of TLRs (2, 3, 4, and 8) in lung ADC from the same patients

**Figure 5:**
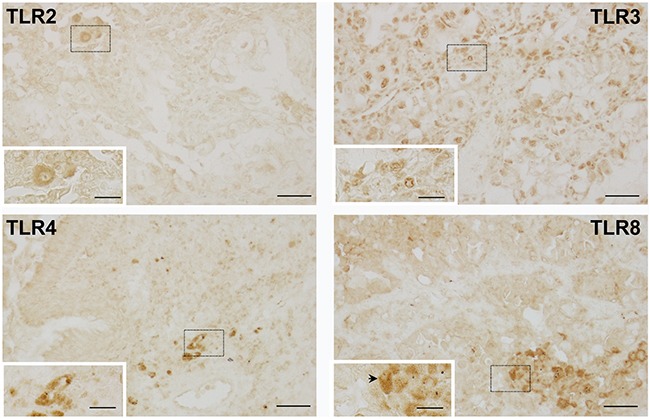
Representative images of TLRs in human neoplastic lung tissue Immunohistochemical staining for TLR's 2, 3, 4 and 8 depicting staining in the tumor cells for all TLRs except TLR8. TLR8 (black arrow) demonstrates macrophage staining. Magnification bars: 100X (black line = 50 μM); insets 200X (black line = 20 μM). Dashed boxes indicate areas magnified within the insets.

These four TLRs were chosen based on significance in the OS analysis and previous studies in our laboratories (TLR4). Positive staining for TLR4 and TLR8 was observed in macrophages around the periphery of the tumors, but sparse staining was observed within the tumor itself (Figure 5). Lymphocytes also stained weakly for TLR4 or TLR8 (data not shown). TLR2 staining (Figure 5) was similar to that observed with TLR4 and TLR8, while TLR3 staining (Figure 5) was observed in all cells.

Analyses were then done to determine any significant correlations between the individual innate immune markers using Metamorph analysis to determine the area stained positively per tissue area (Supplementary Figure 2) followed by Pearson correlations (Table 3). TLR2 expression significantly correlated to TLR4 and TLR8 expression (Table 3). In addition, TLR4 expression also significantly correlated to TLR8 expression. There were no significant differences observed for all of the TLRs immunostained based on tumor staging or morphology, likely due to a low power. However, when stage comparisons were done (data not shown), the data suggests that TLR staining did not vary greatly in the tumors at stages 1-3. Comparisons of staining patterns to inflammatory cell type does support macrophage staining with all TLRs assessed, but not all patients with high numbers of pulmonary macrophages have high TLR staining.

**Table 3:** Correlations between TLRs expression in the tumor tissue.^*+^

Protein	TLR2	TLR3	TLR4	TLR8
**TLR2**	-----------	r^#^ = 0.243	**r = 0.564**	**r = 0.731**
		p<0.331	**p<0.015**	**p<0.0006**
**TLR3**	r^#^ = 0.243	-----------	r = 0.265	r = 0.178
	p<0.331		p<0.288	p<0.480
**TLR4**	**r = 0.564**	r = 0.265	-----------	**r = 0.658**
	**p<0.015**	p<0.288		**p<0.003**
**TLR8**	**r = 0.731**	r = 0.178	**r = 0.658**	-----------
	**p<0.0006**	p<0.480	**p<0.003**	

^*^ n=18 patients for this analysis; + Correlations were performed on the tumor area per tissue areas per protein; ^#^ r = correlation coefficient. Bolded values are significant, p<0.05.

## DISCUSSION

In these studies, we provide evidence for a significant association between high mRNA expression of TLR1-10 in NSCLC patients and improved outcomes for OS (Figure 1) as well as the same comparison for the primary NSCLC subtype, ADC (Figure 2). Interestingly, these findings remain significant when assessed by stage, specifically stage 1. Stage 2 NSCLC and ADC patient numbers were low therefore the analysis while significant for ADC, is less reliable and ≥ stage 3 not evaluated. Thus, a complete analysis of stage was not possible for these studies. Individual analysis for each TLR revealed that high mRNA expression for TLR1, 2, 3, 5, 6, 7, and 8 were significantly associated with improved OS and were likely responsible for the majority of the overall TLR association observed for the analysis. In addition, significant associations between NSCLC patients with high TLR1-10 expression and improved PPS and FP outcomes were also observed. Our analysis of co-expression of the different TLRs also suggests that some of these TLRs are similarly expressed in lung cancer and combined with the OS, PPS, and FP findings, further supports that multiple TLRs increased at the same time could potentially be positive indicators for patients, at least in early stage disease. Molecular alterations in some TLRs may influence response, although the levels of deletions, amplifications, mutations, and mRNA upregulation were generally low in all TLRs (eg. the highest for mRNA upregulation was TLR5 at ∼10%). Thus, these findings in conjunction with the survival analysis further support the concept that increasing TLR expression could improve outcomes. Lastly, we also observed similar positive correlations between TLR4 and 8 when mRNA or protein expression were compared (Table 3). Collectively, these studies demonstrate that TLRs have potential as prognostic markers for lung cancer and further studies to validate these findings are needed. A recent promising study in NSCLC patient serum demonstrated that low soluble TLR4 is a prognostic marker for poor survival of early stage NSCLC [40], supporting TLRs as markers in early stage disease.

Although the expression of these TLRs was similar and some of these significantly correlated to one another, they are expressed in different cellular locations and are activated by different PAMPs. For example, TLR1, 2, 4, 5, 6, and 10 are expressed on the cell surface while TLR3, 7, 8, and 9 are located in endosomal compartments [46]. Receptor dimerization is also different between these TLRs: TLR3, 4, 5, and 9 dimerize with themselves while TLR8 dimerizes with TLR7 and TLR2 dimerizes with itself, TLR1, TLR6, or TLR10 [46, 47]. PAMPs were the ligands initially discovered for these TLRs and most are either bacterial components or viruses, however, endogenous ligands (DAMPs) have been identified that can activate these receptors, such as formyl peptides and heat shock proteins, that are released from injured or dying cells [48]. The ligand(s) that is activating the TLRs in these studies is unclear, however, the similarity in expression between these different TLRs suggests a potential common stimulus inducing endogenous ligands (DAMPs) [18], such as oxidative stress [49] or involvement of chaperones, such as Gp96, an endoplasmic reticulum master chaperone for TLRs [50]. Interestingly, several TLRs are also located at similar chromosomal locations (Supplementary Table 3) and some have transcription factor binding site commonalities. For example, TLR1 and 6 are located on chromosome 4p14 and these two TLRs have a predicted STAT-1 binding site in common (genecards.org). Other TLRs residing on unique chromosomal regions also have transcription factor binding sites in common, such as predicted AP-1/c-Jun sites on TLR1, 2, 3, 4, 5, 6, 7, and 9 (genecards.org). Lastly, based on literature and our staining in lung tissues, these receptors appear to be cell-type specific. This difference in cell type may reflect differences in the ligands and needs to be further evaluated in the future with more clinical samples for each stage to improve our understanding of the cell types involved in regulating these responses. Thus, chromosomal location, transcription factors, cell type, and potential similarities in DAMPs or pathways that activate TLRs may all be contributing factors in the regulation of these TLRs in lung cancer.

The role of the innate immune system in lung cancer remains a controversial issue with respect to pro- or anti-tumorigenic functions and how TLR signaling is involved with tumor cells, epithelial cells, and the tumor microenvironment. We will briefly discuss both pro- and anti-tumorigenic studies in the context of TLRs, but refer readers to a more comprehensive review [42].

The pro-tumorigenic literature for the TLRs demonstrated increased immunostaining of TLRs in NSCLC patients compared to controls for TLR 3, 4, 7, 8, and 9, all in small studies similar to our immunostaining study, which did not differentiate lung cancer subtypes [42, 51, 52]. *In vitro* studies using NSCLC cell lines (A549 and NCI-H1299) demonstrated that TLR4 signaling induced immune escape through immunosuppressive cytokines and resistance to apoptosis [53]. miRNAs have also recently been linked to TLRs in lung cancer in human patients as well as *in vitro* studies in a pro-tumorigenic capacity. TLR4 and TLR9 increase miRNA expression of miR-21 and miR26a, respectively, in a xenograft model or primary human lung cancer cells [54, 55]. One of few TLR1 studies in lung cancer demonstrated miR15a/16 inhibits TLR1 leading to some inhibition of lung tumor xenografts, supporting a role for TLR1 in lung cancer development [56].

As described earlier, our studies using primary mouse lung cancer models have demonstrated a protective nature of TLR4 that resulted in reduced tumor numbers, specific macrophage populations, myeloid-derived suppressor cells (MDSCs), and neutrophils, among other inflammatory cell types [28–33]. Studies in human populations demonstrated that the TLR4 polymorphism that confers reduced TLR activity (rs4986790) was also associated with elevated lung cancer risk [36, 43]. In another human study, elevated levels of TLR5 immunostaining in NSCLC patients associated with an improved diagnosis [45]. Thus, these results support anti-tumorigenic roles for TLRs in tumorigenesis.

Several research groups have tested TLR-targeted therapies in animal models or clinical trials, and although there are risks involved, such as the dual roles of some TLRs, these therapies have some real potential in cancer. We highlight a few of these studies here. Immunomax, a plant-derived agonist for TLR4, is currently in preclinical testing in mice for metastatic breast cancer with prolonged survival as well as decreasing tumor development in 31% of mice [35]. TLR4 is activated via Immunomax turning on dendritic cells, eventually triggering tumoricidal natural killer cells to inhibit tumor growth [35]. Additionally, in breast cancer there are currently several clinical trials assessing efficacy of TLR7/8 agonists (TLR8, VTX-2337 and cyclophosphamide, NCT02650635; TLR7, imiquimod, cyclophosphamide, and radiotherapy) [57–59]. Loxoribin, a TLR7 agonist, inhibited tumor growth using xenograft models for both lung and colon cancer, both mediated by promoting CD4^+^ T cells and activating dendritic cells [39]. Conversely, in A549 cells, TLR7 or TLR8 agonists led to increased tumor cell survival, chemoresistance, and increased anti-apoptotic protein expression [60], as well as appear to increase MDSCs and reduce CD8^+^ T cells in mice, promoting tumor growth [34]. Similar to lung and breast cancer, TLR7 agonists (eg., imiquimod) activate the immune response in skin cancers (melanoma, squamous cell carcinoma, and basal cell carcinoma) and several agonists are currently in clinical trials for melanoma (imiquimod (NCT01678352) and resiquimod (NCT01748747)) [57, 58, 61]. Interestingly, activation of multiple TLRs using a combination of TLR3, TLR4, and TLR7 ligands resulted in tumor rejection in 50% of mice in a B16-OVA tumor model with poor immunogenicity [62], increasing innate immunity and CD8^+^ and CD4^+^ T-cell responses. This report of “multiple adjuvant combination and antigen targeting” [62], supports the use of these TLR ligands in poorly immunogenic tumors and provides a mechanism for why some of these models may be more responsive than others to these TLR ligands. Lastly, two clinical trials using TLR9 agonists (IMO-2055, phase II; MGN1703, phase I) have been utilized as immunomodulators for lung cancer and found possible anti-tumor efficacy, either in combination (IMO-2055) with erlotinib (EGFR inhibitor) and bevacizumab (VEGFA inhibitor) or alone (MGN1703) [37, 41]. MGN1703 is currently in a phase II trial in small cell lung cancer. Based on these trials in lung, the finding that high TLR9 expression in triple negative breast cancer is predictive of an improved prognosis is another area of interest therapeutically [63]. TLR9 expression may influence the tumor immunophenotype and thus has potential to improve chemotherapy and is currently in clinical trials for other types of cancers (eg. lymphomas, colorectal cancer) [58, 59]. We refer readers to several references [57–59] for more comprehensive reviews.

Some limitations of this study are the following. Currently TLR11 does not exist on any of the microarray platforms at cBioportal or kmplot.com, thus could not be evaluated in these studies. The sample size for the TLR immunostaining data set was low, which reduces the statistical power of this study and we only had one pulmonary pathologist review the slides. Also, some clinical data, such as tumor staging was not done for all the tissues. The stage at which lung cancer is detected is crucial to the patient's outcome, e.g. the difference between stage 1 versus stages ≥3 has 5-year survival rates of ∼45% compared to ≤ 14%, respectively. Thus, future experiments should focus on obtaining more samples for these staging analyses.

In conclusion, our novel findings support a role for these TLRs as positive prognostic markers for lung cancer, particularly in early stage disease. More studies are needed to further investigate the addition of these markers into gene panels in current use for early detection and predictive models as well as additional studies in serum of patients similar to the soluble TLR4 study recently published. For example, a combination of identifying TLRs in serum and 18F-FDG positron emission tomography (PET) imaging could aid in determining what patients are sensitive to TLR therapies, similar to other markers (eg p53,[64]). The future of TLRs and their role and possible utility in cancer research will rely on a better understanding of the role of these individual TLRs and the combination of TLRs in the tumor microenvironment, tumor development, and progression, including the specific dimer partners for some TLRs, different types and subtypes of cancer (i.e. SCLC compared to NSCLC), tumor aggressiveness, and the type of specific innate immune cells associated with these TLRs. These findings may be useful for future therapy development and diagnostic strategies for NSCLC.

## MATERIALS AND METHODS

### Meta-analysis for NSCLC and ADC patient survival

We used a program at http://kmplot.com/lung (2015 version; Aug 1, 2016- Sept 1, 2016) for these analyses that uses publically available data sets (Gene Expression Omnibus (GEO); Cancer Biomedical Informatics Grid (caBIG); The Cancer Genome Atlas (TCGA)) from several human Affymetrix microarray platforms because these arrays have 22,277 probe sets in common [65]. The NSCLC patient data sets for 2,427 patients are: GSE4573, GSE14814, GSE8894, GSE19188, GSE3141, GSE31210, GSE29013, GSE37745, GSE30219, GSE31908, GSE43580, GSE50081, caArray, and TCGA (see Győrffy et al., 2013 for clinical characteristics) [65]. Of these data sets, arrays were done on some but not all patients with a total evaluated for NSCLC (OS, n=1926; FP, n=982; PPS, n=344) and the most prevalent subtype of NSCLC, adenocarcinoma (ADC; OS, n=720; FP, n=461; PPS, n=125). Differences in n per TLR evaluated were due to a lack of representation in some data sets, thus the n per TLRs differs for TLR4, 8, and 9. We assessed all 10 TLRs that were present on these Affymetrix platforms; if more than one probe per gene was present, then the Jetset function (http://www.cbs.dtu.dk/biotools/jetset/; Aug. 15, 2016) [66] was used that selects the optimal probe set for each gene. For each TLR, we performed a univariate and multivariate COX regression analysis for OS and FP, and a univariate COX regression analysis for PPS due to a lack of power for both NSCLC and ADC. For the multivariate analysis we used the same co-variates (NSCLC, histology and gender; ADC, stage and gender) as in Győrffy et al., 2013. These specific parameters for the multivariate analyses were chosen where the most clinical information was available. In addition, we also evaluated differences based on stage, however only stage 1 contained sufficient numbers of patients to perform the analysis while stage 2 was preliminary (stage 1, n =449; stage 2, n=161).

Details of the statistical packages used for the development of this program are found in several references [65, 67]. Briefly, the raw CEL files were MAS5 normalized using the Affy Bioconductor library [65, 67]. Quality control excluded biased arrays where 2 or more parameters (ie. percent present calls, background, rawQ, etc) are out of the 95% range of all arrays. Batch effects were also reduced by setting the average expression per chip to 1000 as a second normalization step [65, 67]. The data was then analysed using km-plot as the software that takes R (Bioconductor package within kmplot.com) and the database called PostgreSQL to integrate the gene expression data and clinical data [65, 67]. Kaplan Meier survival plots, the hazard ratio HR with 95% confidence intervals and logrank *P* were then calculated and plotted in kmplot.com stratifying by the median values for gene expression as the threshold for high and low expression per gene evaluated. For the multivariate analyses, each gene was weighted based on 1/HR per gene to account for differences in univariate significance. Lastly, False discovery rate (FDR) was done for multiple testing using kmplot.com (http://kmplot.com/analysis/index.php?p= multiple testing; Aug. 15, 2016) based on the brainwaver library in R [68]. The FDR cutoff was set at 5%. Tumor tissue versus control lung tissue (n=86 samples) from non-tumor-bearing patients was also compared for each TLR via a Mann-Whitney U-test in kmplot.com (Aug. 23, 2016).

### Evaluation of co-expression between TLR1-10 and molecular alterations present in TLR1-10 in ADC patients

The cBioportal database at www.cBioportal.org (Aug. 19, 2016) [69, 70] was used to determine significant co-expression in tumors (mRNA expression from RNAseq in ADC patients) between TLR1-10 via correlation analyses using the TCGA Provisional data set with n=230 patients for the RNAseq analysis [71]. All details on this ADC patient population for these analyses are found in the Supplementary Table 3 and Methods from the Cancer Genome Atlas Research Network (2014; see [71]). Both Pearson and Spearman correlation analyses were performed for all TLRs in cBioportal.org as well as Sigma Plot (12.3). In addition, we identified molecular alterations (amplifications, deletions, mutations, and mRNA upregulation) in ADC patients for each TLR as well as a Pan-Lung Cancer Panel (TCGA, Nature, 2016, [72]) with both ADC (n=585 patients) and squamous cell carcinoma (n=504 patients). (see Supplementary Table 4).

### Patient selection for IHC staining

Eighteen archived lung ADCs were obtained from McLaren Regional Medical Center, Flint, MI (DW). Tumor stage and histopathology was performed by a board certified pathologist (DW). Principle inflammatory cell types were identified by morphology [73]. The clinical characteristics of the tissues used in this study are presented in Table 2, however, because these patients were not part of a clinical trial, we were not able to obtain additional clinical data. All procedures were approved by the Internal Review Board at McLaren (#504) and in accordance with the Biomedical and Health Institutional Review Board at Michigan State University (#08-1114). These studies are in compliance with the Helsinki Declaration, including the additional amendments.

### Immunohistochemistry (IHC) for TLR proteins

Tissue expression of TLR 2, 3, 4, and 8 were evaluated using peroxidase biotin-streptavidin immunohistochemistry. A list of primary antibodies used in this study and dilutions are shown in Supplementary Table 5. Secondary antibodies were purchased from DAKO (Carpinteria, CA) and the remaining reagents from Sigma Chemical Co. (St. Louis, MO) unless otherwise indicated. Lung tissue sections were cut (5 μm per section) (Investigative Histopathology Laboratory, Michigan State University) and then deparaffinized. For antigen retrieval (Supplementary Table 5), slides were steamed for 24-27 minutes. Sections were then treated with 3% H_2_O_2_ to inactivate endogenous peroxidase activity, rinsed, incubated in blocking buffer (2.5% BSA solution prepared in TBST) and then incubated overnight (12 h) at 4°C in primary antibody diluted in blocking buffer. Following incubation, tissues were rinsed with TBST and then treated with DAKO universal labelled streptavidin-biotin2 system, horseradish peroxidase (LSAB2 System HRP) for use with primary antibodies from rabbit or mouse. 3′-3′-diaminobenzidine (DAB) was used for detection. All antibody incubations were performed in Shandon racks (Thermo Fisher Scientific, Waltham, MA) at the same time and sections were washed with TBST between steps.

### Quantification of IHC stain intensity

Tissue-specific expression of the TLRs was quantified using MetaMorph Imaging Software, version 7 (Molecular Devices; Sunnyvale, CA) and a light microscope equipped with a color digitalcamera (Olympus DP20, Tokyo, Japan). Color images (10-20 images per tissue) for each stain were taken under 20X magnification using the auto exposure lock to avoid fluctuations in lighting intensity. Images were then imported into MetaMorph for quantification. For each protein, a color threshold (representing the total pixilated area of positive stain) was set and then quantified. The total tissue area was then measured from grayscale images after thresholding for all-inclusive tissue (total pixilated area of tissue). For each stain assessed, the threshold intensity was kept constant across all images to ensure optimal consistency. Data are expressed as the average area of positive stain in relation to the total surface area of lung tissue. Human tonsil was used as a positive control for TLR antibodies or without the primary antibody, as a negative control (Supplementary Figure 3). Staining intensity was also reviewed and evaluated by DW to examine cell specific expression, ie. tumor versus immune cells.

### Statistical analyses

All statistical analyses, other than those performed in kmplot.com or cBioportal, were conducted using SigmaStat 12.3 (Systat Software, INC, San Jose, CA). Because no significant differences were observed among the tumor stages or tumor morphologies for the IHC staining, all tumor samples and adjacent, non-neoplastic samples were combined for statistics). The correlation analyses were done using the Pearson Correlation, Sigma Stat. For all analyses done, statistical significance was accepted at *P* < 0.05.

## SUPPLEMENTARY MATERIALS FIGURES AND TABLES









## Abbreviations

ADC: Adenocarcinoma
BSA
bovine serum albumin
DAMP: damage-associated molecular pattern
FP: first progression
LPS: lipopolysaccharide, immunohistochemical
OS: overall survival
NSCLC: non-small cell lung carcinoma
PAMP: pathogen-associated molecular patterns
PBS: phosphate buffered solution
PPS: post progression survival
SCLC: small-cell lung carcinoma
TLR: toll-like receptor
TBST: tris-buffered salt solution plus 0.1% tween

## References

[1] Parkin DM, Bray F, Ferlay J, Pisani P (2005). Global cancer statistics, 2002. CA Cancer J Clin.

[2] Schottenfeld D, Pass HI, Mitchell JB, Johnson DH, Turrisi AT, Minna JD (2005). Etiology and Epidemiology of Lung Cancer. Lung Cancer- Principles and Practice.

[3] Bach PB, Jett JR, Pastorino U, Tockman MS, Swensen SJ, Begg CB (2007). Computed tomography screening and lung cancer outcomes. Jama.

[4] Spira A, Beane JE, Shah V, Steiling K, Liu G, Schembri F, Gilman S, Dumas YM, Calner P, Sebastiani P, Sridhar S, Beamis J, Lamb C (2007). Airway epithelial gene expression in the diagnostic evaluation of smokers with suspect lung cancer. Nat Med.

[5] Nowarski R, Gagliani N, Huber S, Flavell RA (2013). Innate immune cells in inflammation and cancer. Cancer Immunol Res.

[6] Bauer AK, Rondini EA (2009). Review paper: the role of inflammation in mouse pulmonary neoplasia. Vet Pathol.

[7] Malkinson AM (2005). Role of inflammation in mouse lung tumorigenesis: a review. Exp Lung Res.

[8] Schottenfeld D, Beebe-Dimmer J (2006). Chronic inflammation: a common and important factor in the pathogenesis of neoplasia. CA Cancer J Clin.

[9] Bauer AK, Dwyer-Nield LD, Hankin JA, Murphy RC, Malkinson AM (2001). The lung tumor promoter, butylated hydroxytoluene (BHT), causes chronic inflammation in promotion-sensitive BALB/cByJ mice but not in promotion-resistant CXB4 mice. Toxicology.

[10] Bauer AK, Dwyer-Nield LD, Keil K, Koski K, Malkinson AM (2001). Butylated hydroxytoluene (BHT) induction of pulmonary inflammation: a role in tumor promotion. Exp Lung Res.

[11] Kumar H, Kawai T, Akira S (2011). Pathogen recognition by the innate immune system. Int Rev Immunol.

[12] Medzhitov R, Preston-Hurlburt P, Janeway CA (1997). A human homologue of the Drosophila Toll protein signals activation of adaptive immunity. Nature.

[13] Poltorak A, He X, Smirnova I, Liu MY, Huffel CV, Du X, Birdwell D, Alejos E, Silva M, Galanos C, Freudenberg M, Ricciardi-Castagnoli P, Layton B (1998). Defective LPS signaling in C3H/HeJ and C57BL/10ScCr mice: mutations in Tlr4 gene. Science.

[14] Medvedev AE, Sabroe I, Hasday JD, Vogel SN (2006). Tolerance to microbial TLR ligands: molecular mechanisms and relevance to disease. J Endotoxin Res.

[15] Akira S, Takeda K (2004). Toll-like receptor signalling. Nat Rev Immunol.

[16] O'Neill LA, Golenbock D, Bowie AG (2013). The history of Toll-like receptors - redefining innate immunity. Nat Rev Immunol.

[17] Peroval MY, Boyd AC, Young JR, Smith AL (2013). A critical role for MAPK signalling pathways in the transcriptional regulation of toll like receptors. PLoS One.

[18] Lagiedo M, Sikora J, Kaczmarek M (2015). Damage-Associated Molecular Patterns in the Course of Lung Cancer--A Review. Scand J Immunol.

[19] Gribar SC, Richardson WM, Sodhi CP, Hackam DJ (2008). No longer an innocent bystander: epithelial toll-like receptor signaling in the development of mucosal inflammation. Mol Med.

[20] Sandor F, Buc M, Toll-like receptors. II (2005). Distribution and pathways involved in TLR signalling. Folia Biol (Praha).

[21] Kleeberger SR, Reddy S, Zhang LY, Jedlicka AE (2000). Genetic susceptibility to ozone-induced lung hyperpermeability: role of toll-like receptor 4. Am J Respir Cell Mol Biol.

[22] Bauer AK, Rondini EA, Hummel KA, Degraff LM, Walker C, Jedlicka AE, Kleeberger SR (2011). Identification of Candidate Genes Downstream of TLR4 Signaling after Ozone Exposure in Mice: A Role for Heat Shock Protein 70. Environ Health Perspect.

[23] Faure K, Sawa T, Ajayi T, Fujimoto J, Moriyama K, Shime N, Wiener-Kronish JP (2004). TLR4 signaling is essential for survival in acute lung injury induced by virulent Pseudomonas aeruginosa secreting type III secretory toxins. Respir Res.

[24] Zhang X, Shan P, Qureshi S, Homer R, Medzhitov R, Noble PW, Lee PJ (2005). Cutting edge: TLR4 deficiency confers susceptibility to lethal oxidant lung injury. J Immunol.

[25] Hollingsworth JW, Whitehead GS, Lin KL, Nakano H, Gunn MD, Schwartz DA, Cook DN (2006). TLR4 signaling attenuates ongoing allergic inflammation. J Immunol.

[26] Astrakianakis G, Seixas NS, Ray R, Camp JE, Gao DL, Feng Z, Li W, Wernli KJ, Fitzgibbons ED, Thomas DB, Checkoway H (2007). Lung cancer risk among female textile workers exposed to endotoxin. J Natl Cancer Inst.

[27] Lundin JI, Checkoway H (2009). Endotoxin and cancer. Environ Health Perspect.

[28] Bauer AK, Dixon D, DeGraff LM, Cho HY, Walker CR, Malkinson AM, Kleeberger SR (2005). Toll-like receptor 4 in butylated hydroxytoluene-induced mouse pulmonary inflammation and tumorigenesis. J Natl Cancer Inst.

[29] Bauer AK, Fostel J, Degraff LM, Rondini EA, Walker C, Grissom SF, Foley J, Kleeberger SR (2009). Transcriptomic analysis of pathways regulated by toll-like receptor 4 in a murine model of chronic pulmonary inflammation and carcinogenesis. Mol Cancer.

[30] Alexander CM, Xiong KN, Velmurugan K, Xiong J, Osgood RS, Bauer AK (2016). Differential innate immune cell signatures and effects regulated by toll-like receptor 4 during murine lung tumor promotion. Exp Lung Res.

[31] Salaun B, Zitvogel L, Asselin-Paturel C, Morel Y, Chemin K, Dubois C, Massacrier C, Conforti R, Chenard MP, Sabourin JC, Goubar A, Lebecque S, Pierres M (2011). TLR3 as a biomarker for the therapeutic efficacy of double-stranded RNA in breast cancer. Cancer Res.

[32] Wang D, Precopio M, Lan T, Yu D, Tang JX, Kandimalla ER, Agrawal S (2010). Antitumor activity and immune response induction of a dual agonist of Toll-like receptors 7 and 8. Mol Cancer Ther.

[33] Zhang Y, Luo F, Cai Y, Liu N, Wang L, Xu D, Chu Y (2011). TLR1/TLR2 agonist induces tumor regression by reciprocal modulation of effector and regulatory T cells. J Immunol.

[34] Dajon M, Iribarren K, Cremer I (2015). Dual roles of TLR7 in the lung cancer microenvironment. Oncoimmunology.

[35] Ghochikyan A, Pichugin A, Bagaev A, Davtyan A, Hovakimyan A, Tukhvatulin A, Davtyan H, Shcheblyakov D, Logunov D, Chulkina M, Savilova A, Trofimov D, Nelson EL (2014). Targeting TLR-4 with a novel pharmaceutical grade plant derived agonist, Immunomax(R), as a therapeutic strategy for metastatic breast cancer. J Transl Med.

[36] Kurt H, Ozbayer C, Bayramoglu A, Gunes HV, Degirmenci I, Oner KS, Metintas M (2016). Determination of the Relationship Between rs4986790 and rs4986791 Variants of TLR4 Gene and Lung Cancer. Inflammation.

[37] Smith DA, Conkling P, Richards DA, Nemunaitis JJ, Boyd TE, Mita AC, de La Bourdonnaye G, Wages D, Bexon AS (2014). Antitumor activity and safety of combination therapy with the Toll-like receptor 9 agonist IMO-2055, erlotinib, and bevacizumab in advanced or metastatic non-small cell lung cancer patients who have progressed following chemotherapy. Cancer Immunol Immunother.

[38] Stevens VL, Hsing AW, Talbot JT, Zheng SL, Sun J, Chen J, Thun MJ, Xu J, Calle EE, Rodriguez C (2008). Genetic variation in the toll-like receptor gene cluster (TLR10-TLR1-TLR6) and prostate cancer risk. Int J Cancer.

[39] Wang C, Zhou Q, Wang X, Wu X, Chen X, Li J, Zhu Z, Liu B, Su L (2015). The TLR7 agonist induces tumor regression both by promoting CD4(+)T cells proliferation and by reversing T regulatory cell-mediated suppression via dendritic cells. Oncotarget.

[40] Wei F, Yang F, Li J, Zheng Y, Yu W, Yang L, Ren X (2016). Soluble Toll-like receptor 4 is a potential serum biomarker in non-small cell lung cancer. Oncotarget.

[41] Weihrauch MR, Richly H, von Bergwelt-Baildon MS, Becker HJ, Schmidt M, Hacker UT, Shimabukuro-Vornhagen A, Holtick U, Nokay B, Schroff M, Wittig B, Scheulen ME (2015). Phase I clinical study of the toll-like receptor 9 agonist MGN1703 in patients with metastatic solid tumours. Eur J Cancer.

[42] Yang LS, Wu WS, Zhang F, Jiang Y, Fan Y, Fang HX, Long J (2014). Role of toll-like receptors in lung cancer. J Recept Signal Transduct Res.

[43] Zhang K, Zhou B, Wang Y, Rao L, Zhang L (2013). The TLR4 gene polymorphisms and susceptibility to cancer: a systematic review and meta-analysis. Eur J Cancer.

[44] Zhao S, Zhang Y, Zhang Q, Wang F, Zhang D (2014). Toll-like receptors and prostate cancer. Front Immunol.

[45] Zhou H, Chen JH, Hu J, Luo YZ, Li F, Xiao L, Zhong MZ (2014). High expression of Toll-like receptor 5 correlates with better prognosis in non-small-cell lung cancer: an anti-tumor effect of TLR5 signaling in non-small cell lung cancer. J Cancer Res Clin Oncol.

[46] Kumar H, Kawai T, Akira S (2009). Toll-like receptors and innate immunity. Biochem Biophys Res Commun.

[47] Govindaraj RG, Manavalan B, Lee G, Choi S (2010). Molecular modeling-based evaluation of hTLR10 and identification of potential ligands in Toll-like receptor signaling. PLoS One.

[48] Lotze MT, Zeh HJ, Rubartelli A, Sparvero LJ, Amoscato AA, Washburn NR, Devera ME, Liang X, Tor M, Billiar T (2007). The grateful dead: damage-associated molecular pattern molecules and reduction/oxidation regulate immunity. Immunol Rev.

[49] Yanagisawa S, Koarai A, Sugiura H, Ichikawa T, Kanda M, Tanaka R, Akamatsu K, Hirano T, Matsunaga K, Minakata Y, Ichinose M (2009). Oxidative stress augments toll-like receptor 8 mediated neutrophilic responses in healthy subjects. Respir Res.

[50] Staron M, Yang Y, Liu B, Li J, Shen Y, Zuniga-Pflucker JC, Aguila HL, Goldschneider I, Li Z (2010). gp96, an endoplasmic reticulum master chaperone for integrins and Toll-like receptors, selectively regulates early T and B lymphopoiesis. Blood.

[51] Chatterjee S, Crozet L, Damotte D, Iribarren K, Schramm C, Alifano M, Lupo A, Cherfils-Vicini J, Goc J, Katsahian S, Younes M, Dieu-Nosjean MC, Fridman WH (2014). TLR7 promotes tumor progression, chemotherapy resistance, and poor clinical outcomes in non-small cell lung cancer. Cancer Res.

[52] Zhang YB, He FL, Fang M, Hua TF, Hu BD, Zhang ZH, Cao Q, Liu RY (2009). Increased expression of Toll-like receptors 4 and 9 in human lung cancer. Mol Biol Rep.

[53] He W, Liu Q, Wang L, Chen W, Li N, Cao X (2007). TLR4 signaling promotes immune escape of human lung cancer cells by inducing immunosuppressive cytokines and apoptosis resistance. Mol Immunol.

[54] Jiang DS, Wang YW, Jiang J, Li SM, Liang SZ, Fang HY (2014). MicroRNA-26a involved in Toll-like receptor 9mediated lung cancer growth and migration. Int J Mol Med.

[55] Zhang X, Wang C, Shan S, Liu X, Jiang Z, Ren T (2016). TLR4/ROS/miRNA-21 pathway underlies lipopolysaccharide instructed primary tumor outgrowth in lung cancer patients. Oncotarget.

[56] Lan F, Yue X, Ren G, Li H, Ping L, Wang Y, Xia T (2015). miR-15a/16 enhances radiation sensitivity of non-small cell lung cancer cells by targeting the TLR1/NF-kappaB signaling pathway. Int J Radiat Oncol Biol Phys.

[57] Gambara G, De Cesaris P, De Nunzio C, Ziparo E, Tubaro A, Filippini A, Riccioli A (2013). Toll-like receptors in prostate infection and cancer between bench and bedside. J Cell Mol Med.

[58] Vacchelli E, Eggermont A, Sautes-Fridman C, Galon J, Zitvogel L, Kroemer G, Galluzzi L (2013). Trial Watch: Toll-like receptor agonists for cancer therapy. Oncoimmunology.

[59] Kang J, Demaria S, Formenti S (2016). Current clinical trials testing the combination of immunotherapy with radiotherapy. J Immunother Cancer.

[60] Cherfils-Vicini J, Platonova S, Gillard M, Laurans L, Validire P, Caliandro R, Magdeleinat P, Mami-Chouaib F, Dieu-Nosjean MC, Fridman WH, Damotte D, Sautes-Fridman C, Cremer I (2010). Triggering of TLR7 and TLR8 expressed by human lung cancer cells induces cell survival and chemoresistance. J Clin Invest.

[61] Hari A, Flach TL, Shi Y, Mydlarski PR (2010). Toll-like receptors: role in dermatological disease. Mediators Inflamm.

[62] Aranda F, Llopiz D, Diaz-Valdes N, Riezu-Boj JI, Bezunartea J, Ruiz M, Martinez M, Durantez M, Mansilla C, Prieto J, Lasarte JJ, Borras-Cuesta F, Sarobe P (2011). Adjuvant Combination and Antigen Targeting as a Strategy to Induce Polyfunctional and High-Avidity T-Cell Responses against Poorly Immunogenic Tumors. Cancer Res.

[63] Sandholm J, Selander KS (2014). Toll-like receptor 9 in breast cancer. Front Immunol.

[64] Hasbek Z, Dogan OT, Sari I, Yucel B, Seker MM, Turgut B, Berk S, Silig Y (2016). The Diagnostic Value of the Correlation between Serum Anti-p53 Antibody and Positron Emission Tomography Parameters in Lung Cancer. Mol Imaging Radionucl Ther.

[65] Gyorffy B, Surowiak P, Budczies J, Lanczky A (2013). Online survival analysis software to assess the prognostic value of biomarkers using transcriptomic data in non-small-cell lung cancer. PLoS One.

[66] Li Q, Birkbak NJ, Gyorffy B, Szallasi Z, Eklund AC (2011). Jetset: selecting the optimal microarray probe set to represent a gene. BMC Bioinformatics.

[67] Szasz AM, Lanczky A, Nagy A, Forster S, Hark K, Green JE, Boussioutas A, Busuttil R, Szabo A, Gyorffy B (2016). Cross-validation of survival associated biomarkers in gastric cancer using transcriptomic data of 1,065 patients. Oncotarget.

[68] Gyorffy B, Gyorffy A, Tulassay Z (2005). The problem of multiple testing and solutions for genome-wide studies [Article in Hungarian]. Orv Hetil.

[69] Cerami E, Gao J, Dogrusoz U, Gross BE, Sumer SO, Aksoy BA, Jacobsen A, Byrne CJ, Heuer ML, Larsson E, Antipin Y, Reva B, Goldberg AP (2012). The cBio cancer genomics portal: an open platform for exploring multidimensional cancer genomics data. Cancer Discov.

[70] Gao J, Aksoy BA, Dogrusoz U, Dresdner G, Gross B, Sumer SO, Sun Y, Jacobsen A, Sinha R, Larsson E, Cerami E, Sander C, Schultz N (2013). Integrative analysis of complex cancer genomics and clinical profiles using the cBioPortal. Sci Signal.

[71] Cancer Genome Atlas Research Network (2014). Comprehensive molecular profiling of lung adenocarcinoma. Nature.

[72] Campbell JD, Alexandrov A, Kim J, Wala J, Berger AH, Pedamallu CS, Shukla SA, Guo G, Brooks AN, Murray BA, Imielinski M, Hu X, Ling S (2016). Distinct patterns of somatic genome alterations in lung adenocarcinomas and squamous cell carcinomas. Nat Genet.

[73] Ross MH, Pawlina W (2006). Respiratory System. Histology: A text and atlas with correlated cell and molecular biology.

