# Supplementary material for: Toll-like receptor expression in human non-small cell lung carcinoma: potential prognostic indicators of disease
Source: Oncotarget. 2017 Jul 22;8(54):91860–75. doi: 10.18632/oncotarget.19463 (PMC5696147; doi:10.18632/oncotarget.19463)
Supplement: Supplementary file 1 [file oncotarget-08-91860-s001.pdf]

Toll-like receptor expression in human non-small cell lung carcinoma: potential prognostic indicators of disease

SUPPLEMENTARY MATERIALS

Supplemental Methods

Cell maintenance

NCI-H1650 cells were cultured in RPMI-1640 (Invitrogen) containing 10% FBS and 1% glutamate in a humidified atmosphere at 37˚ C, 5% CO_2, and 95% air. HBE1 cells were a kind gift from Dr. Reen Wu (University of California Davis). HBE1 cells were cultured in the following defined media: Hams F12 media (Invitrogen) containing 10 µg/ml insulin (Sigma), 1 µM hydrocortisone (Sigma), 3.75 µg/ml Endothelial Cell Growth Supplement (Sigma), 25 ng/ml EGF (Sigma), 5 µg/ml transferrin (Sigma), and 10 ng/ml Cholera toxin (Sigma), similar to Yankaskas et al, 1993 [1]. Both cell lines were grown to confluence and then RNA prepared.

Quantitative reverse transcriptase PCR for TLRs 1-10

RNA was isolated from the cells using Macherey-Nagel Nucleospin RNA II kit (Clontech Laboratories, Mountain View, CA) following their kit specifications. cDNA was prepared using oligo-dT, followed by PCR amplification in 50 µl reaction volumes [2]. qRT-PCR was then done using the cDNA stock and human gene specific primers (intron-spanning) with KAPA SYBR FAST mastermix (Kapa Biosystems; Boston, MA) on a Mastercycler EP Realplex[4] qRT-PCR cycler (Eppendorf; Hauppauge, NY) [3]. Primer sequences for *HPRT1* and *TLR1-10* are found in [4, 5]. HPRT1 is preferred as a normalizer gene for NSCLC [4].

REFERENCES

1. Yankaskas JR, Haizlip JE, Conrad M, Koval D, Lazarowski E, Paradiso AM, Rinehart CA Jr7, Sarkadi B, Schlegel R, Boucher RC. Papilloma virus immortalized tracheal epithelial cells retain a well-differentiated phenotype. Am J Physiol. 1993; 264: C1219-30.
2. Bauer AK, Fostel J, Degraff LM, Rondini EA, Walker C, Grissom SF, Foley J, Kleeberger SR. Transcriptomic analysis of pathways regulated by toll-like receptor 4 in a murine model of chronic pulmonary inflammation and carcinogenesis. Mol Cancer. 2009; 8: 107.
3. Hill T 3rd, Osgood RS, Velmurugan K, Alexander CM, Upham BL, Bauer AK. Bronchoalveolar Lavage Fluid Utilized Ex Vivo to Validate *In Vivo* Findings: Inhibition of Gap Junction Activity in Lung Tumor Promotion is Toll-Like Receptor 4-Dependent. J Mol Biomark Diagn. 2013; 5. doi: 10.4172/2155-9929.1000160.
4. Gresner P, Gromadzinska J, Wasowicz W. Reference genes for gene expression studies on non-small cell lung cancer. Acta Biochim Pol. 2009; 56: 307-16.
5. Kanuri G, Ladurner R, Skibovskaya J, Spruss A, Konigsrainer A, Bischoff SC, Bergheim I. Expression of toll-like receptors 1-5 but not TLR 6-10 is elevated in livers of patients with non-alcoholic fatty liver disease. Liver Int. 2015; 35: 562-8. doi: 10.1111/liv.12442.

SUPPLEMENTARY FIGURES AND TABLES

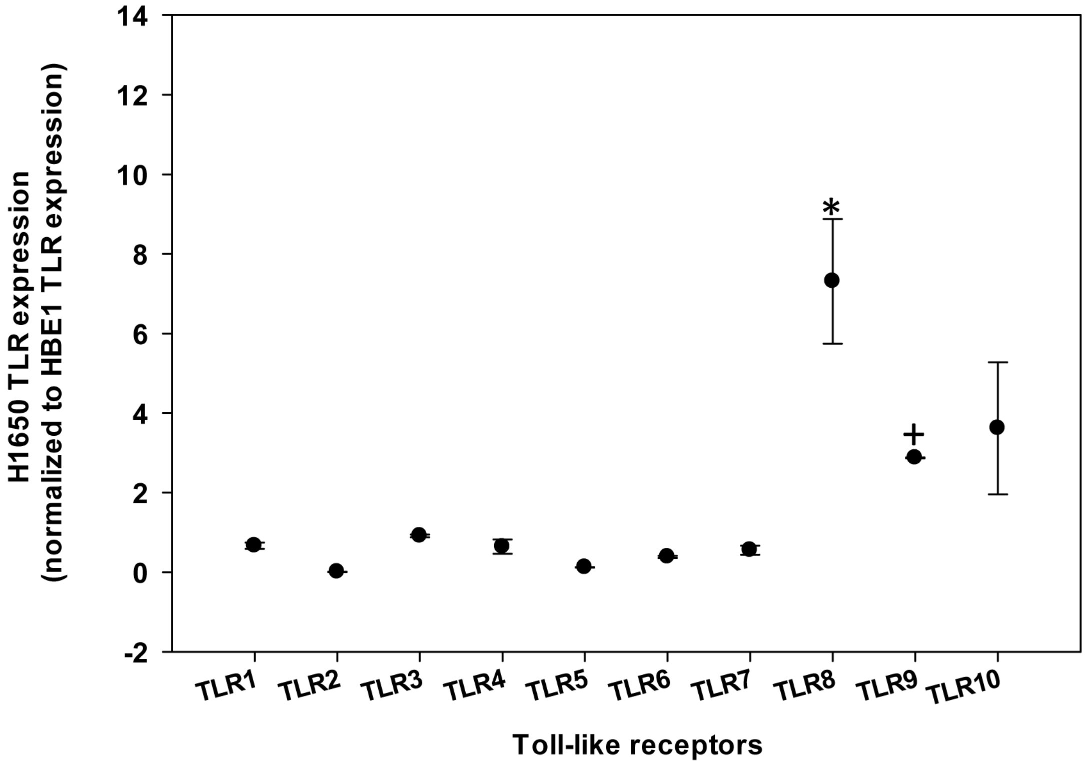

Supplementary Figure 1: TLR 1-10 mRNA expression in NCI-H1650 cells. Quantitative RT-PCR was performed to investigate TLR 1-10 in a human adenocarcinoma cell line (NCI-H1650). All samples were first normalized to *HPRT1* followed by comparison to HBE1 cells, a normal human bronchial epithelial cell line. TLRs 1-7 were similar in mRNA expression, however TLRs 8, 9, and 10 were more variable compared to the HBE1 cells. Mean ± SEM, repeated once. *, $P<0.05$ for TLR8 compared to TLR1, 2, 3, 4, 5, 6, 7 and 9. +, $P<0.05$ for TLR9 compared to TLR1, 2, 3, 4, 5, 6, and 7.

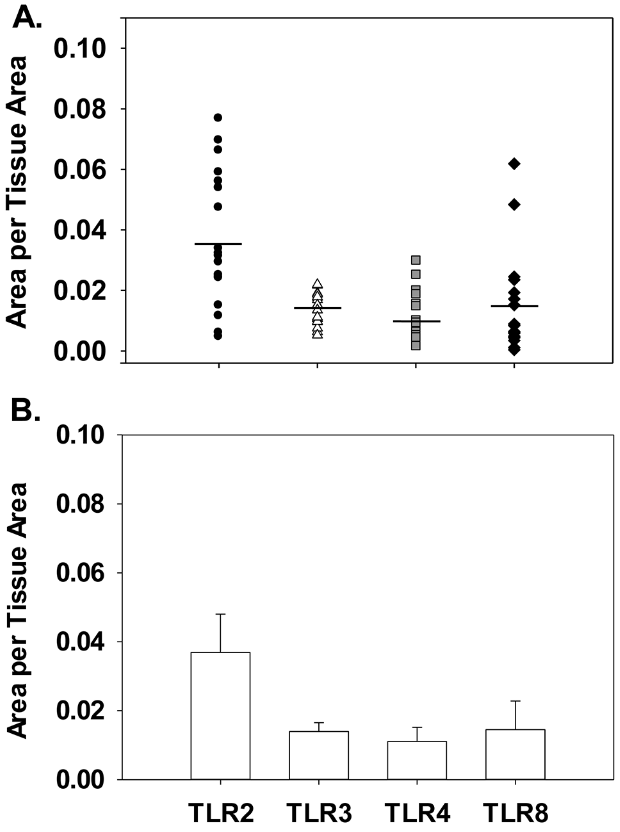

Supplementary Figure 2: Quantification of TLRs 2, 3, 4, and 8 in ADC. Immunohistochemical staining for TLR's 2, 3, 4, and 8 were quantified using Metamorph Imaging Software and digital images. **(A)** Data are expressed as mean area of stain per total tissue area for each patient. Tumor stages and morphologies were combined. Mean is denoted with a horizontal line. **(B)** Same data graphed as the mean ± 95% confidence intervals for the mean area of stain per total tissue area (n=18 patients).

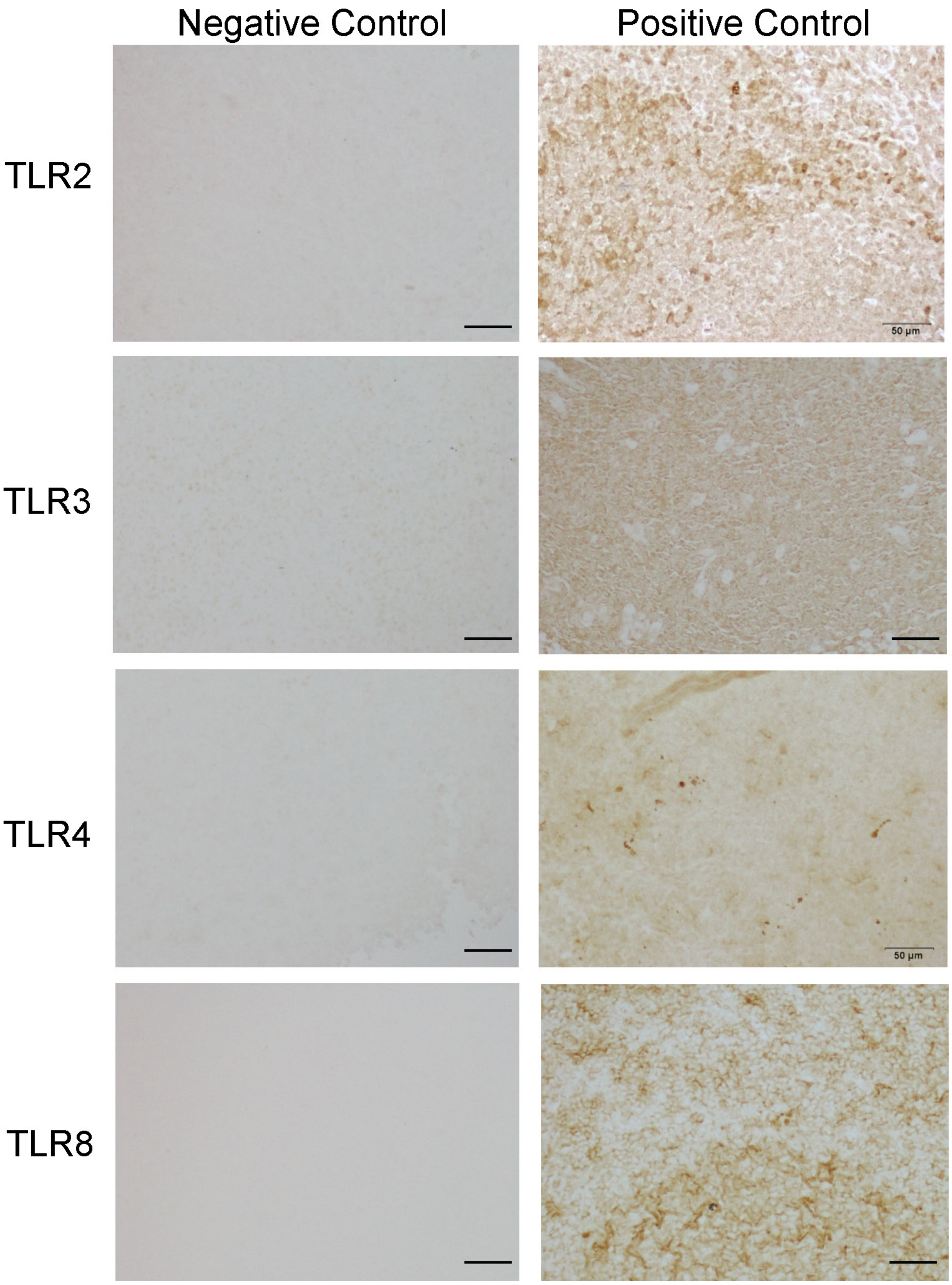

Supplementary Figure 3: Human Tonsil was used as a control tissue for TLR staining. Sections were stained without primary antibody as a negative control. Pictures were taken with an Olympus BX41 at 20X. Scale bar = 50 µM.

Supplementary Table 1: Data from meta-analysis using kmplot.com for all TLRs for both NSCLC and ADC. (A) NSCLC and OS meta-analysis; (B) ADC and OS meta-analysis.

See Supplementary File 1

Supplementary Table 2: Analysis of TLR expression in control patient's lung tissue compared to NSCLC patient's tumor tissue. Control (n=86) and tumor tissue from NSCLC patients (n=1926) was compared and Mann-Whitney U tests performed.

TLR	Probe ID	Tissue	Min	Quartile 1	Median	Quartile 3	Max	*p* value**	n
1	210176_at	Normal	2	23	124	242	856		86
1	210176_at	Cancer	6	140	227	346	1916	**2.8E-09**	1926
2	204924_at	Normal	3	71	374	1081	4302		86
2	204924_at	Cancer	3	404	729	1291	8267	**4.8E-06**	1926
3	206271_at	Normal	1	47	191	302	562		86
3	206271_at	Cancer	1	102	196	366	2827	**0.02**	1926
4	232068_at	Normal	0	18	86	178	729		86
4	232068_at	Cancer	1	44	77	126	1270	NS	1587
5	210166_at	Normal	2	54	378	568	1223		86
5	210166_at	Cancer	9	216	382	471	1667	NS	1926
6	239021_at	Normal	4	29	54	98	223		86
6	239021_at	Cancer	1	33	54	89	506	NS	1926
7	220146_at	Normal	1	12	68	148	398		86
7	220146_at	Cancer	1	59	112	224	1617	**7.4E-07**	1926
8	229560_at	Normal	1	72	338	645	1663		86
8	229560_at	Cancer	3	206	377	673	5228	NS	1587
9	223903_at	Normal	6	47	86	121	334		86
9	223903_at	Cancer	2	40	60	93	807	**.00633**	1587
10	223751_at	Normal	1	23	58	108	237		86
10	223751_at	Cancer	2	42	72	127	1976	**0.003**	1926

*Control lung tissue from non-tumor bearing patients; **p value for tumor compared to normal tissue determined via a Mann-Whitney analysis; **bolded** *p* values are significant.

Supplementary Table 3: Co-expression of TLRs with other genes in ADC patients. (A) all genes co-expressing with TLR1 in ADC patients; (B) all TLR genes co-expressing with TLR1 in ADC patients; (C) all genes co-expressing with TLR2 in ADC patients; (D) all TLR genes co-expressing with TLR2 in ADC patients; (E) all genes co-expressing with TLR3 in ADC patients; (F) all TLR genes co-expressing with TLR3 in ADC patients; (G) all genes co-expressing with TLR4 in ADC patients; (H) all TLR genes co-expressing with TLR4 in ADC patients; (I) all genes co-expressing with TLR5 in ADC patients; (J) all TLR genes co-expressing with TLR5 in ADC patients; (K) all genes co-expressing with TLR6 in ADC patients; (L) all TLR genes co-expressing with TLR6 in ADC patients; (M) all genes co-expressing with TLR7 in ADC patients; (N) all TLR genes co-expressing with TLR7 in ADC patients; (O) all genes co-expressing with TLR8 in ADC patients; (P) all TLR genes co-expressing with TLR8 in ADC patients; (Q) all genes co-expressing with TLR9 in ADC patients; (R) all TLR genes co-expressing with TLR9 in ADC patients; (S) all genes co-expressing with TLR10 in ADC patients; (T) all TLR genes co-expressing with TLR10 in ADC patients. Pearson correlations are computed first. For genes with a correlation greater than 0.3 or less than -0.3, the Spearman correlations are also computed, all in cBioportal.com. n=230 ADC patients analyzed via RNA sequencing (see [71]).

See Supplementary File 2

Supplementary Table 4: Molecular alterations present in TLRs in ADC patients. (A) pan-lung cancer genes altered organized by gene, total percentage of patients (cases) altered, somatic mutation (%), deletions (%), and amplifications (%); (B) types of genetic alterations in pan-lung cancer patients; (C) ADC genes altered organized by gene, total percentage of patients (cases) altered, somatic mutation (%), deletions (%), amplifications (%), and mRNA upregulation (%); (D) types of genetic alterations in ADC patients. Pan-lung cancer panel (n=1144 patients) [72]; ADC patients (n=230) [71].

See Supplementary File 3

Supplementary Table 5: Summary of primary antibodies used for immuno histochemistry.

Antibody	Company	Catalog No.	Species/Clonality	Concentration/ Dilution used	Antigen Retrieval Buffer*
TLR2	Imgenex	IMG-319	Mouse IgG1, monoclonal	1:35 dilution	Citrate
TLR3	Imgenex	IMG-315A	Mouse IgG1, monoclonal	5 µg/mL	Tris-EDTA
TLR4	Abcam	Ab47093	Rabbit IgG, polyclonal	10 µg/mL	Tris-EDTA
TLR8	Imgenex	IMG-321A	Mouse IgG1, monoclonal	2 µg/mL	Tris-EDTA

* All antigen retrieval was conducted with steam heat using a steamer. The composition of buffers was either 10 mM Citrate (pH 6.0) or 10 mM Tris containing 1 mM EDTA (pH 9.0).
